# Exploring the efficacy and tolerability of two‐injection start regimen of long‐acting aripiprazole: A descriptive case series analysis

**DOI:** 10.1002/npr2.12489

**Published:** 2024-10-12

**Authors:** Ibrahim Sungur, Kaan Keskin, Elif Özge Aktaş, Mehmet Çağdaş Eker

**Affiliations:** ^1^ Department of Psychiatry Ege University Izmir Turkey; ^2^ SoCAT Lab Ege University Izmir Turkey

**Keywords:** antipsychotics, aripiprazole, bipolar disorder

## Abstract

Bipolar disorder is the fourth most debilitating psychiatric illness in the world regarding Disability Adjusted Life Years and manic episodes frequently lead to lengthy hospitalizations which restricts the freedom of patients. Therefore, decreasing the length of hospitalization with safer agents is of utmost importance in the treatment of manic episodes. Aripiprazole is a medication known for its efficacy in managing mania associated with bipolar disorder. Aripiprazole long‐acting injection is approved for the treatment of mania associated with bipolar disorder in adults and found efficacious as a maintenance treatment. In the treatment of schizophrenia, European Medicines Agency has approved a simplified starting strategy of aripiprazole once a month, with two 400 mg injections and a single oral 20 mg dose of aripiprazole. To the best of our knowledge, no previous study has reported the safety, tolerability, and efficacy of this regimen in adult bipolar disorder patients. We present a case series of eight patients who were admitted to the hospital in a manic episode with psychotic features. We observed that the double injection start regimen was effective in treating manic symptoms with no specific severe adverse events. We conclude from a small sample of manic patients that a double injection start regimen has good efficacy and tolerability.

## Abstract 2

Evaluate the safety, tolerability, and efficacy of the TIS regimen of Aripiprazole in treating manic episodes in bipolar disorder. Retrospective observational study involving eight hospitalized patients with bipolar disorder in a manic episode.

## Graphical abstract

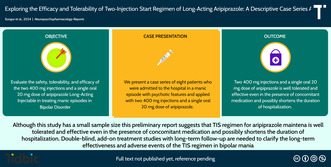

## 1 BACKGROUND

Bipolar disorder (BD) is the fourth most debilitating psychiatric illness globally in terms of Disability Adjusted Life Years.^1^ Manic episodes often lead to lengthy hospitalizations, restricting patient freedom. In 2002, the average hospitalization duration was 47 days, costing €22 297, with 98.6% due to hospitalization. In 2022, mental health‐related costs for manic/mixed episodes were $15 444 for inpatient and $1577 for outpatient settings.^2^, ^3^ Moreover, for the treatment of repeated agitation multiple sedatives and typical neuroleptics are used for a long time which is enough to lead to adverse events due to treatment. Therefore, decreasing the length of hospitalization with safer agents is of utmost importance in manic episodes.

Aripiprazole is effective for managing mania in bipolar disorder.^4^ Calabrese et al. found Aripiprazole long‐acting injection (LAI) effective for both acute and maintenance treatment of BD, leading to Food and Drug Administration approval for its maintenance use.^5^, ^6^ Recently, the European Medicines Agency has approved a simplified starting strategy of aripiprazole LAI with two 400 mg injections and a single oral 20 mg dose of aripiprazole administered on the same day for the treatment of schizophrenia.^7^

We report the safety, tolerability, and efficacy of this regimen in adult BD manic episode patients in the real world with a case series of eight patients who were admitted to the hospital in a manic episode and treated with aripiprazole LAI one‐day start dose regimen. We discuss the effectiveness and safety of this two‐injection start (TIS) regimen of aripiprazole long‐acting injectable in 1‐day with concomitant medications.

## 2 MATERIALS AND METHODS

This retrospective observational study aimed to evaluate the safety and tolerability of the aripiprazole once a month (AOM) 400 mg TIS regimen in a consecutive cohort of patients who started AOM according to the dosing guidelines in the prescribing information^7^ from December 2022 to March 2024. The study was approved by the Ege University Ethics Committee (number 24‐3T/36, approval date March 7, 2024) and it conformed to the provisions of the Declaration of Helsinki.

Patients were included according to the following inclusion criteria.

1. Age ≥ 18 years.
2. Diagnosis of BD, manic episode.
3. Initiation of AOM according to the TIS regimen.

The safety of the TIS regimen was retrospectively evaluated through patient files and interviews with patients and attending physicians. Sociodemographic, clinical, and safety data were extracted from clinical records by trained psychiatrists using the hospital's electronic patient data system. We scanned patient files and interviewed patients for any reported physical symptoms or adverse effects. Serious/severe adverse effects were defined as those potentially related to any medication that resulted in death, required hospitalization or prolonged hospitalization, were life‐threatening, or resulted in significant disability/incapacity. Patients were routinely administered the Young Mania Rating Scale (YMRS) and Montgomery–Åsberg Depression Rating Scale (MADRS) on the first day of hospitalization, at the initiation of the TIS regimen, and on the 10th day of the regimen.

## 3 RESULTS

This study included eight patients who received the TIS regimen. Demographic and clinical features are shown in Table 1. All patients were in the manic episode and all required hospitalization because of the severity of the illness. As can be seen in Table 1 all patients had psychotic and mixed features. The severity of the episode requiring hospitalization was determined by the level of functional impairment, risk of harm to self or others, or high severity of the acute episode, as indicated by high scores on both the YMRS and MADRS.

**TABLE 1:** Demographic and clinical features.

Case no.	Age/sex	Duration of current episode (days)	Psychotic features (delusions/hallucinations)	Number of prior manic/depressive episodes	Number of hospitalizations	Number of suicide attempts	Age at onset/duration of illness (years)	Comorbidity	Family history
1	53/Male	Pre: 15 Host: 20 Total: 35	Persecutory/jealousy/reference delusions	Unknown	0	0	38/15	Hypertension	AD
2	24/Female	Pre: 30 Host: 28 Total: 58	Persecutory/mystic/reference delusions	0/1	0	1	23/1	AUD	None
3	48/Male	Pre: 60 Host: 32 Total: 92	Persecutory/reference delusions	2/2	2	0	23/25	Diabetes Mellitus, Hypertension	None
4	41/Female	Pre: 90 Host: 4 Post: 8 Total: 102	Persecutory/jealousy delusions	5/1	4	0	17/24	None	None
5	37/Female	Pre: 43 Host: 18 Total: 61	Grandiose/reference delusions Auditory hallucination	2/0	1	0	27/10	None	SCZ
6	21/Male	Pre: 30 Host: 36 Total: 66	Persecutory delusions	2/0	1	0	15/6	Hypothyroidism	BD^a^
7	38/Male	Pre: 10 Host: 22 Total: 32	Bizarre/grandiose/ persecutory/reference delusions	2/4	3	0	24/13	OCD, GAD	BD
8	29/Male	Pre: 30 Host: 16 Total: 46	Grandiose delusions	3/0	1	0	27/2	Hypertension CUD	GAD

*Note*: Pre: episode duration before the hospitalization, Host: episode duration in the hospital, Post: episode duration after the hospitalization, Total: total duration of current episode.

Abbreviations: ALZ, Alzheimer's disease; AUD, alcohol use disorder; BD, bipolar disorder; CUD, cannabis use disorder; GAD, general anxiety disorder; OCD, obsessive compulsive disorder; SCZ, schizophrenia.

^a^ Two family members are diagnosed with BD.

We treated the patients described in the study based on published guidelines for the treatment of manic and mixed episodes with psychotic features and based on our clinical expertise. We prescribed oral benzodiazepines and IM (intramuscular) typical antipsychotics (e.g., haloperidol or zuclopenthixol) for agitation because IM benzodiazepines and atypical antipsychotics are not available in our country. To avoid physical restraints, we used typical antipsychotics and benzodiazepines alongside mood stabilizers for agitated patients. In three cases,^2^, ^6^, ^7^ we prescribed clozapine because of the ineffectiveness of other antipsychotics and the need to avoid extrapyramidal side effects and severe sedation from high doses of other antipsychotics. The TIS regimen choice was based on the severity of symptoms and prior non‐adherence history.

The mood symptom scale measurements and physical symptoms due to treatment are presented in Table 2.

**TABLE 2:** Progression of mood symptoms and adverse events.

Case no.	Initiation day of TIS regimen	1st day of TIS regimen YMRS‐MADRS‐psychotic features	1st day of TIS regimen concomitant medication	10th day of post‐ TIS YMRS‐MADRS‐psychotic features	10th day of post‐TIS regimen concomitant medication	Post‐ TIS regimen time to full remission (days)	Initiation day of TIS regimen adverse effects	Post‐TIS 10th day adverse effects	Total duration of hospitalization (days)
1	4th	YMRS:39 MADRS:22 Persecutory/jealousy/ reference delusions	AAP+.BZD+ CPZ:875	YMRS:19, MADRS:8, No psychotic features	AAP+ BZD+CPZ: 600	16	Dysarthria ‐Tachycardia	‐Sedation ‐Dizziness ‐Dysarthria ‐Tachycardia	20
2	5th	YMRS:59 MADRS:18 Persecutory/mystic/ reference delusions	AAP+ BZD+ TAP+ MS+ CPZ:2125	YMRS:20, MADRS:7, No psychotic features	AAP+ BZD+TAP+ MS+CPZ: 2700	24	‐None	‐Sedation ‐Tachycardia ‐Hypersalivation ‐Constipation	29
3	6th	YMRS:48 MADRS:21 Persecutory/reference delusions	AAP+ BZD+ TAP+ MS+ CPZ:2375	YMRS:22, MADRS:6, Persecutory reference delusions	AAP+ BZD+TAP+ MS+CPZ: 2100	27	‐Muscular Rigidity	‐Sedation ‐Muscular Rigidity	33
4	1st	YMRS:55 MADRS:16 Persecutory/jealousy delusions	AAP+.BZD+ CPZ:875	YMRS:3, MADRS:2, No psychotic features	AAP+.BZD+MS+CPZ: 300	12	‐None	‐Sedation ‐Dysarthria ‐Fever ‐Appetite increase	4
5	1st	YMRS:41 MADRS:14 Grandiose/reference delusions Auditory hallucination	AAP+ TAP+ CPZ:450	YMRS:12, MADRS:2, No psychotic features	AAP+ MS+CPZ: 875	18	‐None	‐Sedation	20
6	22nd	YMRS:26 MADRS:14 Persecutory delusions	AAP+ BZD+ TAP+ MS+ CPZ:2725	YMRS:4, MADRS:1, No psychotic features	AAP+ BZD+TAP+ MS+CPZ: 2300	11	‐Sedation	‐Sedation	39
7	1st	YMRS:53 MADRS:19 Bizarre/grandiose/ persecutory/reference delusions	AAP+ BZD+ TAP+ CPZ:1975	YMRS:26, MADRS:10, Persecutory/reference thoughts	AAP+ BZD+TAP+CPZ: 2000	22	‐None	‐Tachycardia ‐Sedation ‐Hypersalivation ‐Sexual side effects	26
8	1st	YMRS:50 MADRS:22 grandiose delusions	AAP+ MS+ TAP+ CPZ:1375	YMRS:10, MADRS:2, No psychotic features	AAP+ MS+TAP+CPZ: 3225	16	‐None	‐Sedation ‐Rash	23

Abbreviations: AAP, atypical antipsychotics; BZD, benzodiazepines; CPZ, chlorpromazine equivalent dose; MADRS, Montgomery–Åsberg Depression Rating Scale; MS, mood stabilizers; TAP, typical antipsychotic; TIS regimen, two‐injection of aripiprazole long‐acting injectable in one‐day dose regimen; YMRS, Young Mania Rating Scale.

In clinical practice, we rely on detailed daily examinations and observations rather than scales for side effects. Our daily electronic records capture all follow‐up information. There were two unexpected adverse events: Patient 4 experienced a sub‐febrile fever, which responded to an antipyretic and did not recur. Patient 6 developed a unilateral hand rash, which resolved within 24 h. We have shown the clinical effects of the TIS regimen in Table 3.

**TABLE 3:** Clinical effects of two‐injection of aripiprazole long‐acting injectable in 1‐day dose regimen.

Mean day of TIS regimen initiation	Mean YMRS score on 1st day of TIS regimen	Mean YMRS score on 10th day of post‐TIS regimen	Mean time to remission post‐TIS regimen (days)	Mean total duration of hospitalization (days)
5.10 (±6.66)	46.30 (±9.95)	14.50 (±7.97)	18,25 (±5.31)	24.50 (±6.63)^a^

Abbreviations: TIS regimen, two‐injection of aripiprazole long‐acting injectable in 1‐day dose regimen; YMRS, Young Mania Rating Scale.

^a^ Patient number four was excluded because the patient and family decided to refer to outpatient care.

## 4 DISCUSSION

In this case series, we aimed to present our observations on the TIS regimen for adult BD patients. To the best of our knowledge, there are no case series on the TIS regimen in adult BD patients regarding effectiveness or adverse effects, though three studies involve schizophrenia patients and an adolescent BD patient. These studies reported good efficacy and tolerability with no severe adverse effects.^8^, ^9^, ^10^ Previous reports on AOM 400 mg for BD in placebo‐controlled trials primarily noted akathisia, weight gain, insomnia, and anxiety as treatment‐emergent adverse events. Cuomo et al. (2023) observed predominantly akathisia and tremor in schizophrenia patients starting the TIS regimen.^10^ However, we observed sedation, tachycardia, and hypersalivation the most. This discrepancy with previous reports may be due to our small sample size and the use of concomitant medications. Therefore, randomized studies with larger groups are needed to confirm our results.

Although AOM is effective in preventing BD relapses, it requires oral supplementation for the first 2 weeks, potentially increasing relapse risk in non‐compliant patients. A recent population pharmacokinetic analysis supports the TIS regimen to reduce reliance on daily oral administration and optimize AOM's therapeutic benefits in schizophrenia patients.^11^ The TIS regimen may improve adherence at treatment initiation, shorten time to remission in manic episodes, and reduce hospitalization duration, allowing for earlier discharge.

Longer durations of mania and hospitalization lead to increased episode severity and frequency, worse clinical outcomes, higher re‐hospitalization rates, increased healthcare costs, and negative impacts on functioning. Hospitalization times for manic episodes range from 5.6 to 41.2 days,^12^ and a Turkish study found durations of 12–34 days.^13^ Moreover, many patients were not in remission at discharge, which predicts relapse within a year and affects functionality.^14^

Hospitalizations also bring interpersonal and financial problems, along with a restriction of freedom. The World Psychiatric Association recommends outpatient treatment whenever possible.^15^ Shortening hospitalization or treating patients in outpatient settings aligns with mental health organization recommendations and reduces treatment costs. In the United Kingdom, the annual National Health Service cost for bipolar disorder is 342 million pounds, with 60% due to hospitalizations.^16^ Safely reducing hospitalization duration would allow resources to be reallocated to other areas of mental health care in need.

We observed that six out of eight patients achieved full remission within 3 weeks following the TIS regimen and the remaining two within 4 weeks. Sooner the administration of the TIS regimen sooner the patients were ready for discharge and, hence the application of the TIS regimen determined the duration of hospitalization in most of the cases.

Five of the eight patients were free of psychotic symptoms by day 10 of the TIS regimen, which is remarkable given the severity of their manic episodes. However, all patients also received high doses of other antipsychotics, likely contributing to this outcome. Our observation was that the response to the TIS regimen was not immediate, becoming apparent only by the end of the first week. By then, we noticed a rapid recovery of manic symptoms and increased cooperation, followed by partial remission by day 10. Initially, we were hesitant to reduce antipsychotic doses early in the treatment. However, reviewing these cases has given us more confidence in tapering concomitant medication doses by the end of week one and stopping all antipsychotics by day 10 for future applications.

This case series is inherently retrospective, with a small sample size (*N* = 8), limiting the generalizability of the findings. The clinical characteristics of the patients, such as severe manic episodes with psychotic and mixed features and the use of concomitant medications, may have also influenced the observed responses and side effects. These limitations highlight the need for larger randomized controlled trials to validate our findings. Additionally, there is potential for recall bias since the data were gathered from past medical records, which may not always accurately reflect patient medication and follow‐up. Although we relied on meticulous record‐keeping practices, recall bias cannot be entirely ruled out. Since all cases were inpatients with detailed daily observations and records, we believe that recall bias is mitigated but not completely eradicated.

Since the primary aim was to stabilize patients with the most effective and safe treatment, not to test a novel approach, we also used mood stabilizers, antipsychotics, and benzodiazepines as needed. This makes it difficult to determine whether the observed effectiveness and adverse events were due to the TIS regimen, other medications, or both. The retrospective design and lack of a control group prevent us from firmly establishing the efficacy of the TIS regimen. Future studies should include a control group that does not follow the TIS regimen to establish a stronger causal relationship between the intervention and the outcome.

Additionally, the use of concomitant medications complicates the interpretation of the TIS regimen's efficacy and tolerability, further highlighting the need for controlled studies. Typical efficacy and tolerability studies often exclude severe patients with comorbidities and prohibit concomitant medications, raising questions about the real‐world effectiveness and safety of trial medications. In this real‐world study with severe patients, we found that the TIS regimen is safe when used with concomitant medications. However, caution should be exercised when evaluating our results regarding side effects and adverse events, as we did not use scales to assess extrapyramidal side effects.

This case series does not include follow‐up data, although all cases continue treatment in our outpatient unit. The absence of follow‐up data to assess long‐term benefits and potential delayed adverse effects is a limitation. Double‐blind, add‐on treatment studies with long‐term follow‐up are needed to clarify the long‐term effectiveness and adverse events of the TIS regimen in bipolar mania.

Due to the small sample size and design, statistical methods beyond descriptive statistics were impractical. We found the mean YMRS score decreased from 46.30 (±9.95) on the first day, indicating a severe episode, to 14.50 (±7.97) after 10 days, indicating partial remission. The mean time to remission following the TIS regimen was 18.25 (±5.31) days, which is relatively short given the severity and psychotic‐mixed features of the episodes.

We report eight severe mania cases with psychotic and mixed features treated with the TIS regimen of aripiprazole Maintena and concomitant medications. Despite the small sample, this preliminary report suggests that the TIS regimen is well tolerated, effective, and may shorten hospitalization duration.

## AUTHOR CONTRIBUTIONS

Ibrahim Sungur, Kaan Keskin, Elif Özge Aktaş, and Mehmet Çağdaş Eker conceptualized and wrote the manuscript. Kaan Keskin, Elif Özge Aktaş, and Mehmet Çağdaş Eker did the clinical evaluation and rating scale administration. Ibrahim Sungur and Mehmet Çağdaş Eker were involved in the literature search and revisions. All authors read and approved the final manuscript.

## FUNDING INFORMATION

The authors did not receive any specific funding for this work.

## CONFLICT OF INTEREST STATEMENT

The authors declare no conflict of interest.

## ETHICS STATEMENT

Approval of the Research Protocol by an Institutional Reviewer Board: The study was approved by the Ege University Ethics Committee (number 24‐3T/36, approval date March 7, 2024).

Informed Consent: The patients have consented in written form to the submission of the case report for submission to the journal.

Registry and the Registration No. of the Study/Trial: N/A.

Animal Studies: N/A.

## Data Availability

Data sharing is not applicable to this article as no new data were created or analyzed in this study.
